# Morphological and nutritional responses of sorghum to variable irrigation levels and nitrogen doses

**DOI:** 10.1371/journal.pone.0323901

**Published:** 2025-06-02

**Authors:** Beyza Ciftci, Ihsan Serkan Varol, Sevim Akcura, Yusuf Murat Kardes, Safa Karaman, Mahmut Kaplan

**Affiliations:** 1 Department of Field Crops, Faculty of Agriculture, University of Erciyes, Kayseri, Türkiye; 2 Department of Biosystems Engineering, Faculty of Agriculture, University of Erciyes, Kayseri, Türkiye; 3 Department of Field Crops, Faculty of Agriculture, University of Canakkale Onsekiz Mart, Canakkale, Türkiye; 4 Department of Field Crops, Faculty of Agriculture and Natural Science, University of Bilecik Seyh Edebali, Bilecik, Türkiye; 5 Department of Food Engineering, Faculty of Engineering, Nigde Omer Halis Demir University, Nigde, Türkiye; KGUT: Graduate University of Advanced Technology, IRAN, ISLAMIC REPUBLIC OF

## Abstract

This study aimed to determine the effects of different irrigation levels (50%, 75%, and 100% of ETo values calculated using evaporation from Class-A pan) and nitrogen doses (0, 90, 180, and 270 kg ha ⁻ ¹) on yield, yield components, and the nutritional properties of sorghum grains. According to the research results, increasing irrigation and nitrogen fertilization levels enhanced plant height, thousand-grain weight, grain number per panicle, grain weight per panicle, and grain yield. The highest grain yield (7120 kg ha ⁻ ¹) was obtained with 100% irrigation and 180 kg ha ⁻ ¹ N application. While increasing irrigation levels increased oil content, higher nitrogen doses caused a decrease for it. The highest oil content (6.64%) was recorded with 100% irrigation and 0 kg ha ⁻ ¹ N application. Protein content increased with irrigation and nitrogen applications, reaching the highest level (11.85%) with 100% irrigation and 270 kg ha ⁻ ¹ N application. Higher irrigation levels also increased total starch and phytic acid content. Among nitrogen applications, the dose of 270 kg ha ⁻ ¹ resulted in the maximum total starch (77.29%) and phytic acid content (1.83%). The ratio of resistant starch (RS) was found to be high at 50% irrigation with low nitrogen doses, indicating an inverse relationship with the total starch content. Both irrigation and nitrogen applications significantly affected the ratios of oleic and linoleic acids. Specifically, increased irrigation raised the linoleic acid content, while nitrogen applications enhanced the oleic acid content. Additionally, as irrigation levels increased, the contents of potassium (K), magnesium (Mg), iron (Fe), phosphorus (P), and zinc (Zn) also increased. Conversely, the levels of calcium (Ca) and manganese (Mn) decreased. Generally, higher nitrogen doses resulted in increased mineral content, with the highest levels of magnesium, iron, and zinc observed at nitrogen doses between 180 and 270 kg ha ⁻ ¹.

According to the research results, the most suitable irrigation level for optimizing high yield and grain nutritional properties was determined to be 100%, with a nitrogen dose of 180–270 kg ha ⁻ ¹. These findings will contribute to future studies on different sorghum varieties under varying climate and soil conditions.

## Introduction

Sorghum (*Sorgum bicolor*) grain is a staple food for millions of people in developing countries as it contains carbohydrates, protein, fat, and minerals. Recently, it has also become recognized as a functional food of choice worldwide [1,2]. Sorghum is a significant ingredient in ensuring food safety and is utilized as a substitute for wheat to produce gluten-free foods including couscous, pasta, bread, cookies, breakfast cereals, and even alcoholic and non-alcoholic beverages like vinegar [3]. Sorghum is a crop that is rich in carbohydrates and has the potential to be a highly nutritious grain for human consumption [4]. In fact, it is the fifth most important cereal crop in the world, following wheat, corn, rice, and barley [5]. The physical, chemical, thermal, and rheological properties of sorghum starch are heavily influenced by the ratio of amylose to amylopectin in the grain [6]. The functionality of starch, such as gelatinization, retro gradation, dough viscosity, and gelation, is influenced by amylose and amylopectin content [7]. Sorghum grains contain resistant starch, which is a dietary fraction that cannot be digested in the intestines [8]. The phytic acid content of cereal grains can reduce amino acid content and protein digestibility through the complexes they form. Although sorghum grain is rich in Mg, Fe, Zn, Cu, Ca, P, and K, phytic acid can also reduce the bioavailability of important mineral cations such as Fe, Zn, and Ca [9].

The quality of products made from sorghum is directly linked to the biochemical structure of sorghum itself [10]. The biochemical properties of sorghum grain are influenced by genetic traits as well as agricultural practices like irrigation and fertilization, as highlighted in the research conducted by Kardes et al. [11] and Kaplan [12]. Irrigation and fertilization are both the leading agricultural costs and the most important factors affecting yield and quality [13]. Excessive water and fertilizer use, in addition to cost, also causes negative effects on the soil and the environment and causes productivity losses. Nitrogen is an essential element for the growth, development, and yield of plants. It plays a vital role in various morphological and metabolic processes of plants, including nutrient uptake, antioxidant activities, photosynthesis, and respiration [14]. However, its excessive use in intensive agricultural production can lead to soil acidification, an increase in reactive nitrogen components in the environment, and a change in the nitrogen structure of the soil, resulting in ecosystem degradation [15]. Therefore, it is crucial to apply nitrogen at an appropriate rate to regulate plant growth and yield and prevent nitrogen pollution [16]. Climate change has caused changes in the amount and distribution of precipitation, leading to a negative impact on water resources and their dynamics [17]. However, while water demand is rising, water resources are also decreasing due to climate change. The high temperatures, intense light, and dry wind caused by climate change have not only limited water resources but also led to drought -[18]. Drought stress can cause significant changes in the morphological and physiological characteristics of plants, such as root development, plant height, stem diameter, and leaf amount [13]. Additionally, properties like mineral content, carbohydrate amount, and protein properties in plants vary with the amount of irrigation [19,20]. Drought stress is considered a significant threat to crop production [21] and is therefore extensively studied by researchers around the world.

Despite the critical importance of irrigation and nitrogen in sorghum production, their effects on growth characteristics, grain yield, and nutritional properties of the grain remain widely unknown. The aim of this study was to determine the effects of different irrigation levels and nitrogen doses on morphological characteristics, grain yield and nutritional properties of sorghum.

## Materials and methods

The Akdarı sorghum variety, which is an early-maturing type characterized by a large panicle, white seeds, and an elliptical seed structure, was used as the research material to examine the effect of three different irrigation levels (IW50: 50%, IW75: 75%, and IW100: 100% of the ET_0_ value calculated from the evaporation measured from the Class A evaporation container) and four different nitrogen doses (0, 90, 180 and 270 kg ha^-1^ N) on some nutritional profile of the samples was investigated. The trial was conducted in the trial area of Çanakkale Onsekizmart University Faculty of Agriculture, using a divided plots trial pattern, and the trial was planted on April 20, 2020, and April 25, 2021. The seeds were sown over 5 x 4.2 m plots with 70 cm row spacing and 15 cm on-row plant spacing.

Trial irrigation levels were established as the main parcel, and nitrogen doses were applied as the sub-plot. Nitrogen dose was applied in the form of urea. The randomized blocks were separated into plots with three replications, and a distance of 2 meters was maintained between the plots to prevent any fertilizer doses from transferring between the irrigation levels. Soil analysis determined nitrogen doses, which were applied in two phases - half at the time of planting and half when the plants reached a height of 40–50 cm. Additionally, 180 kg/ha^-1^ P_2_O_5_ was applied at planting. In the study, only P₂O₅ and nitrogen fertilization were applied, with no additional fertilization treatments. The nitrogen was mixed into the soil at planting and top fertilization was provided with irrigation water. At maturity stage, harvesting was done manually, and threshing was carried out using a threshing machine. Weed control was done manually and no chemical spraying was applied. No disease was observed in the plants. In field experiment, plant height, thousand seed weight, number of grains per panicle, weight of grains per panicle and seed yield were examined.

### Soil and climate characteristics of the experimental area

Soil samples were collected from four depth intervals (0–30, 30–60, 60–90, and 90–120 cm) and analyzed at the Erciyes University Laboratory. The results of the analyses are summarized in Table 1. The soil texture was predominantly clay-loam, except for the 60–90 cm layer, which was classified as sandy-clay-loam. Saturation ranged from 39.90% to 59.40%, while field capacity (32.5–36.2%) and permanent wilting point (19.8–22.6%) exhibited slight variations. Bulk density remained consistent across depths (1.39–1.46 g cm ⁻ ³), and available water content slightly decreased with depth (56–54 mm). The pH values (7.78–8.16) indicated a neutral to slightly alkaline profile. Electrical conductivity (0.48–1.47 dS m ⁻ ¹) and lime content (12.01–18.1%) increased with depth. Organic matter was highest at 60–90 cm (1.91%) and lowest at 90–120 cm (0.82%). Phosphorus content declined with depth (475.8–285.9 kg P₂O₅ ha ⁻ ¹), whereas potassium levels peaked at 30–60 cm (864.8 kg K₂O ha ⁻ ¹).

**Table 1:** Soil analysis results of the trial areas.

Properties	Depth (cm)			
	0–30	30–60	60–90	90–120
Texture	Clay-Loam	Clay-Loam	Sandy-Clay-Loam	Clay-Loam
Saturation (%)	48.93	59.4	40.30	39.90
pH	7.78	8.08	8.11	8.16
EC (dS m^-1^)	0.48	0.435	0.85	1.470
Lime (%)	12.01	18.1	14.8	14.3
Organic Matter (%)	1.49	1.74	1.91	0.82
Phosphorus (kg P_2_O_5_ ha^-1^)	475.8	396.3	386.2	285.9
Potassium (kg K_2_O ha^-1^)	621.7	864.8	–	–
FC (%)	32.8	36.2	35.1	32.5
PWP (%)	19.8	22.6	22.5	19.9
BD (gr cm^-3^)	1.43	1.39	1.46	1.44
AW (mm)	56	57	55	54

**EC** electrical conductivity; **FC:** field capacity; **PWP:** permanent wilting point; **BD:** bulk density; **AW:** available water.

The climate parameters of the experimental site are presented in Table 2. The data used in the experiment were obtained from Station No. 17112 of the Çanakkale Meteorology Directorate. A comparison between long-term climate data (1937–2019) and the experimental years (2020 and 2021) highlights significant differences in temperature, relative humidity, and precipitation patterns. The mean temperature during the experimental years exceeded the long-term average, with 2021 recording the highest mean (25.1°C) compared to 22.5°C. Maximum temperatures also increased, peaking at 33.8°C in July 2021. Relative humidity exhibited a decreasing trend, particularly in 2021, where it declined to an average of 57%, compared to 66.8% in the long-term data. Precipitation patterns varied; while total precipitation in 2020 (96.7 mm) surpassed the long-term average (72.6 mm), it was significantly lower than in 2021 (116.4 mm). Evaporation rates were elevated during the experimental years, with 2020 reaching 1006.9 mm, indicating increased water loss. (Table 2).

**Table 2:** Long-term and experiment years averages of climate parameters in Çanakkale province.

Months	Temperature (°C)			Relative Humidity (%)	Wind Speed (m·s^ − 1^)	Rainfall (mm)	Evaporation (mm)
	Min	Mean	Max				
**Long term (1937–2019)**							
May	12.8	17.6	22.6	73.2	3.4	32.0	168.3
June	16.7	22.3	27.8	67.6	3.3	22.4	217.0
July	19.4	25.1	30.7	62.9	3.8	11.7	268.3
August	19.6	25.0	30.6	63.3	4.0	6.5	252.2
September	15.9	20.9	26.4	68.0	3.7	24.2	170.8
Mean/Total	17.1	22.5	27.9	66.8	3.6	72.6	905.8
**2020 year**							
May	13.5	18.2	23.6	68.9	3.0	54.6	151.9
June	17.8	22.6	28.4	74.0	3.0	38.8	213.7
July	21.7	27.0	32.9	55.3	4.1	0.1	341.2
August	21.6	27.1	33.4	54.2	3.5	3.2	300.1
September	20.6	24.7	29.7	59.6	3.5	9.5	229.9
Mean/Total	18.7	23.7	29.6	63.1	3.4	96.7	1006.9
**2021 year**							
May	16.0	19.9	25.2	66.6	3.1	57.3	181.7
June	19.5	24.1	29.3	58.3	2.0	57.1	193.0
July	23.5	28.2	33.8	52.0	2.7	2.0	314.5
August	24.1	28.3	33.4	51.1	2.3	0.0	289.8
September	19.1	23.1	27.6	54.0	2.7	8.9	233.7
Mean/Total	20.8	25.1	30.4	57.0	2.5	116.4	979.0

### Irrigation system and irrigation treatments

Irrigations were practiced through drip lines. Irrigation treatments were set as 50%, 75% and 100% of ETo value calculated with the use of weekly evaporations from Class-A evaporation pan. System control units included pressure regulator, fertilizer tank, sand-gravel filter, sieve filter, manometers, valves and water meters. For water distribution, main, manifold and lateral lines and drippers were used. Irrigation water was supplied from a deep-well located close to the experimental fields. The Irrigation water quality class was C3S1 with a SAR value of 6.44, an electrical conductivity value of 882 µmhos/cm and a pH value of 7.6. Lateral lines were 16 mm in diameter, dripper flow rate was 1.6 l/h and dripper spacing was 70 cm. Lateral lines were placed on each sorghum row, and a water meter was placed at the entrance of each block.

Drip irrigation method was used in this study. In the first irrigation, soil moisture was brought to field capacity. Afterward, equal amounts of irrigation water were applied to all treatments until sufficient root development. After the plants achieved sufficient development, irrigation treatments were initiated. The amount of irrigation water to be applied in each irrigation treatment was determined by using evaporations from Class-A pan placed in the trial area. Weekly total evaporations from open-water surface were converted into reference evapotranspiration and 50%, 75% and 100% of these converted values were applied. The equation suggested by [22] was used to calculate the amount of irrigation water to be applied.


ETo = E pan x Kpan

where;

ETo: Reference crop evapotranspiration

Epan: Pan evaporation

Kpan: Pan coefficient (For the Class-A evaporation pan, Kpan varies between 0.35–0.85, it was taken as 0.70)

### Crop water consumptions (ET)

The water budget equation given in [23] was used to determine crop water consumptions:


ET= I+R+Cr−Dp−Rf±Δs

where;

ET: Crop water consumption (mm), I: Irrigation water quantity (mm), R: Effective precipitation (mm), Cr: Capillary rise (mm), Dp: Deep percolation (mm), Rf: Surface runoff (mm), ∆s: Change in soil moisture (mm).

### Irrigation water productivity

Irrigation water use efficiency (IWP) values were calculated with the use of the following equations [24].


$$IWP\;=\frac{Y}{I}$$

where;

IWUE: Irrigation water productivity (kg ha^-1^ mm^-1^),

Y = Yield (kg ha^-1^),

I = Amount of irrigation water applied (mm).

### Crop water productivity

Crop water productivity (CWP) is defined differently by different researchers [25]. CWP could be defined as the production quantity or value per unit of consumed or diverted water. It is calculated as the ratio of actual yield to the volume of water utilized:


$$CWP\;=\;\frac{Y}{ETa}$$

where;

CWP = Crop water productivity (kg ha^-1^ mm^-1^),

Y = Yield (kg ha^-1^)

ETa = Actual evapotranspiration (mm)

### Biochemical assay

Harvested sorghum grains were dried at 60 °C, ground (IKA MF-10.1, Staufen, Germany), and made ready for biochemical analyses. Sorghum grain samples were preserved at -20 °C throughout the analysis.

### Crude protein content

About 1 g of sample was taken and the nitrogen ratio was determined with the use of the macro Kjeldahl method. The resultant nitrogen value was multiplied by 6.25 (N x 6.25) to get sample crude protein content [26].

### Crude ash content

About 1 g of sample was carbonized in a muffle furnace at 550 ºC for 8 hours to get the crude ash content of the samples.

### Non-resistant, resistant, and total starch content

Megazyme Resistant Starch Assay (catalog number K-RSTAR, Megazyme International Ireland Ltd. Co. Wicklow, Ireland) kit developed in accordance with AOAC Official Method 2002.02 and AACC 32–40 Method was used to determine resistant starch content of 100 mg samples.

### Amylose-Amylopectin content

Amylose and amylopectin fractions of the starch were determined using the Megazyme Amylose/Amylopectin Analysis Kit (K-AMYL, Megazyme International Ireland, Wicklow, Irelan).

### Total dietary fiber content

Total dietary fiber analysis of the samples will be performed using the Megazyme total dietary fiber kit (AOAC 991.43, AACC 32–07.01, AOAC 985.29 and AACC 32–05.01) and will be calculated as (K-TDFR) and % dietary fiber content.

### Pythic acid content

Phytic acid content was detected with phytic acid assay kit (K-PHYT, Megazyme Intl, Wicklow, Ireland) in accordance with the instructions of the manufacturer. About 1 g sample was used in the analysis.

### Mineral contents

Sorghum whole grain samples were subjected to acid digestion in nitric perchloric acid [27] and then P, Mg, K, Ca, Fe, Na, S, Mn, Cu Zn, and B contents were determined in an ICP-OES spectrometer (Inductively Couple Plasma spectrophotometer) (Agilent 5800) [28].

### Crude oil and fatty acid composition

Seed samples (3 g) were dissolved with ether in a Soxhlet cartridge. Oil-extracted samples were then kept in a drying chamber at 95 °C for an hour, cooled in a desiccator and ether extract values were calculated with the aid of an equation [26]. Fatty acids in the lipid extracts were converted into methyl esters by means of 2% sulphuric acid (v/v) in methanol [29]. The fatty acid methyl esters were extracted with n-hexane. Then the methyl esters were separated and quantified by gas chromatography and flame ionization detection (Shimadzu GC, 17 Ver.3) coupled with a glass GC 10 software computing recorder. Chromatography was performed with a capillary column (25m in length and 0.25mm in diameter, Permabound 25, Machery-Nagel, Germany) using nitrogen as carrier gas (flow rate 0.8mL/min), the temperatures of the column, detector and injector valve were 130–220, 240–280 °C, respectively. Identification of the individual method was performed by frequent comparison with authentic standards mixtures that were analyzed under the same conditions.

### Data analysis

First of all, variance analysis was performed over the combined years with the use of JMP SAS statistical software [30]. Significant means were compared with the use of LSD multiple comparison test with the alpha level was 0.01.

## Results

In 2020 and 2021, the irrigation water levels corresponding to 50%, 75%, and 100% treatments were 324, 486, and 648 mm, and 312, 468, and 624 mm, respectively. In 2020, evapotranspiration ranged from 414 mm to 791 mm, while in 2021, it varied between 398 mm and 763 mm. It was observed that an increase in nitrogen doses led to higher ET (except I50 × N18) by the crop (Table 3). The highest average CWP was determined to be 12.41 kg ha^-1^ mm^-1^, while the lowest was 5.83 kg ha^-1^ mm^-1^. The average IWP ranged from 7.08 to 15.22 kg ha^-1^ mm^-1^ (Table 3).

**Table 3:** Evapotranspiration, CWP and IWP values of sorghum.

I (%)	N (kg ha^-1^)	ET (mm/season)			CWP (kg ha^-1^ mm^-1^)			IWP (kg ha^-1^ mm^-1^)		
		2020	2021	Mean	2020	2021	Mean	2020	2021	Mean
**50**	**0**	414	398	406	6.14	6.16	6.15	7.84	7.86	7.85
**50**	**90**	459	401	430	10.19	9.14	9.67	14.44	11.75	13.09
**50**	**180**	404	394	399	12.41	11.86	12.14	15.46	14.98	15.22
**50**	**270**	419	405	412	10.61	11.42	11.01	13.72	14.82	14.27
**75**	**0**	588	572	580	6.98	5.83	6.40	8.44	7.13	7.78
**75**	**90**	591	560	576	8.15	8.35	8.25	9.92	9.99	9.95
**75**	**180**	583	570	577	10.94	10.75	10.84	13.12	13.09	13.11
**75**	**270**	597	581	589	9.24	8.19	8.71	11.35	10.16	10.76
**100**	**0**	768	751	760	5.86	6.00	5.93	6.95	7.22	7.08
**100**	**90**	782	748	765	7.30	8.07	7.69	8.81	9.68	9.25
**100**	**180**	779	741	760	9.68	9.04	9.36	11.63	10.74	11.19
**100**	**270**	791	763	777	8.02	7.74	7.88	9.79	9.46	9.63

**Fig. 1:**
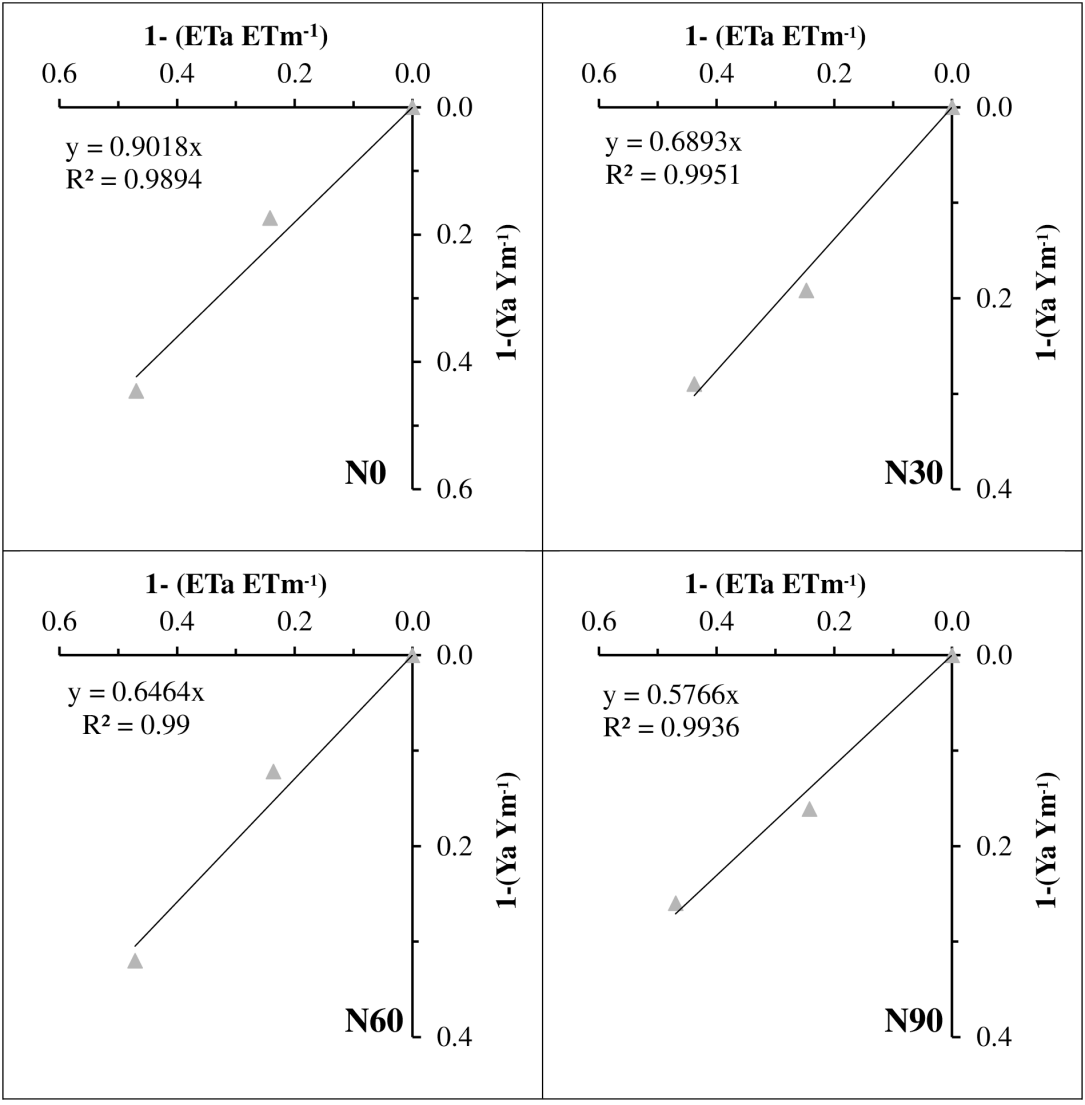
Yield response factors of sorghum.

The yield response factor (ky) was calculated for all nitrogen doses based on each average value and R^2^ values were determined. The value, which was 0.90 in 0 ha^-1^ N dose, became more tolerant in 30 ha^-1^ N application and was calculated as 0.69. Ky, which was 0.65 in 60 kg ha^-1^ N application, was determined as 0.57 in 90 ha^-1^ N dose. As indicated by the high R² values, there is a significant response between water and yield. Moreover, the increase in nitrogen doses made the crop more sensitive to irrigation (Fig 1).

**Fig 2:**
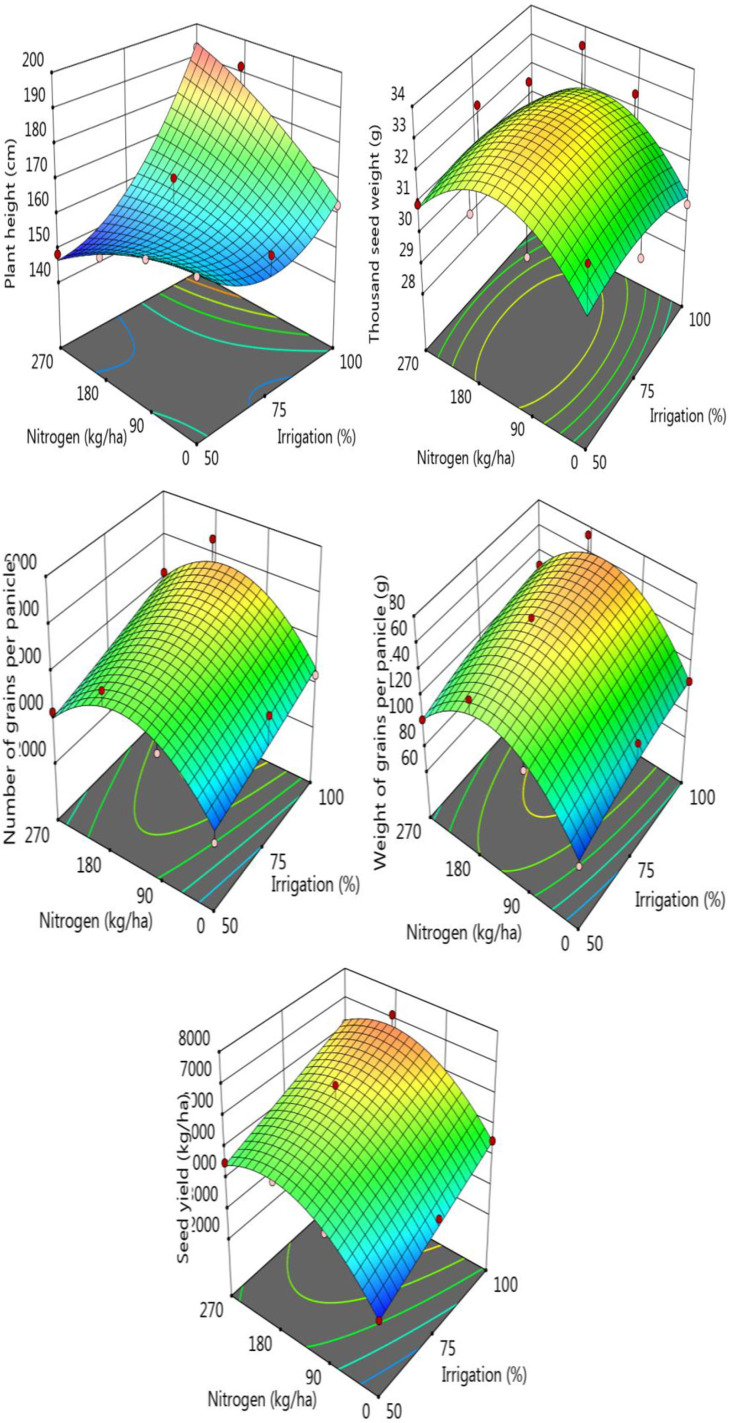
Change in seed yield and some morphological parameters of the samples according to the nitrogen-irrigation treatments.

The changes in morphological characteristics and seed yield of sorghum samples in response to different water and nitrogen treatments are given in Supplementary Information Table 1, Fig 2. Plant height of the samples showed a significant increase in parallel with the increase in irrigation level, while plant height increased again with the increase of nitrogen addition at constant water level. The average plant height was recorded as 157.46 cm for the 50% irrigation level while the height of plant was 180.50 cm for the 100% irrigation treatment. Increase in the nitrogen level, plant height values also increased. The thousand grain weight values varied between 30.50 and 32.91 g and the thousand grain weight values, which were independent of the increase in irrigation amount, also increased with the of increasing nitrogen level. Averaged number of grains per panicle were in the range of 3221.85–4197.97 for irrigation and 2962.81–4552.42 for the nitrogen treatments (S1 File). Increase in the irrigation level increased the number of grains per panicle significantly and similar observations were also recorded for the samples treated with different doses of nitrogen. The average weight of grains per panicle ranged between 109.45–136.57 g for the irrigation levels and 96.52–113.42 g for the nitrogen treatments. Increase in the irrigation resulted in a significant increase in the weight of grains per panicle as similar to the nitrogen treatment. Grain yield values are also affected by both irrigation levels and nitrogen doses significantly. Increase in irrigation, grain yield increased from 4011 to 5906 kg ha^-1^ while the increase in nitrogen level from 0 to 180 kg ha^-1^, grain yield increased from 3573 to 6071 kg ha^-1^. The highest grain yield of 7120 kg ha^-1^ was obtained from 100% irrigations level and 180 kg ha^-1^ nitrogen dose application (S1 File, Fig 2).

**Fig 3:**
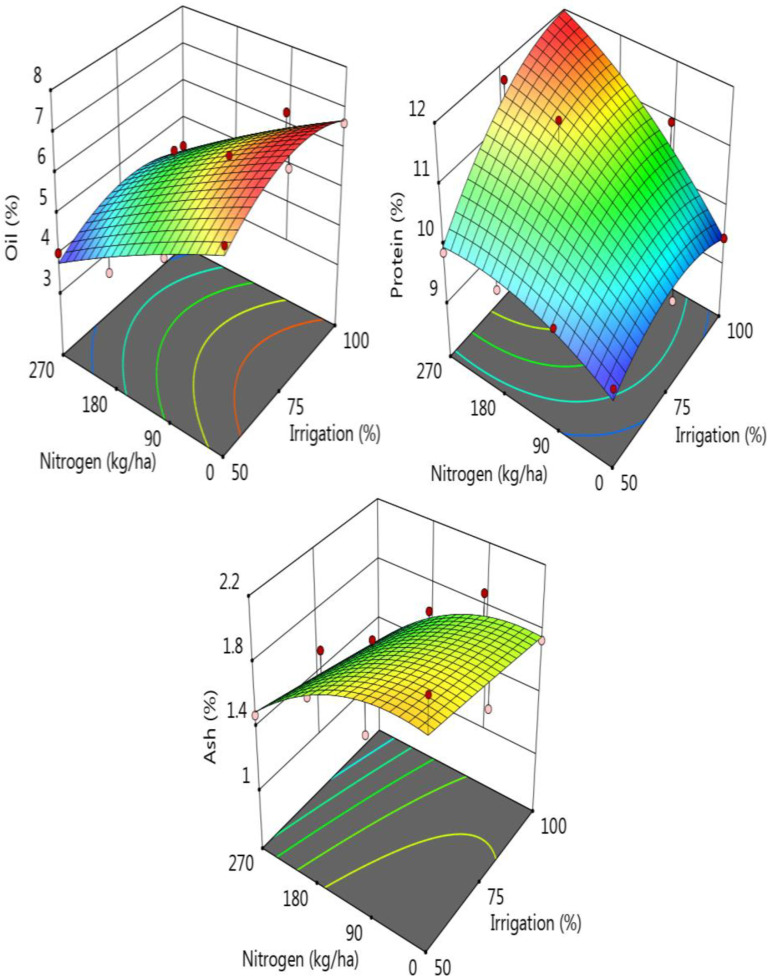
Change in some proximate parameters of the samples according to the nitrogen-irrigation treatments.

Fig 3 and S2 File shows the biochemical characteristics of the sorghum samples exposed to different irrigation levels and nitrogen doses. Oil content of the sorghum samples increased with irrigation and significantly decreased with the increase in nitrogen doses (Fig 3). The highest oil content was recorded for 75% irrigation and the lowest oil level was monitored for the sample treated with 270 kg ha^-1^ nitrogen. On the contrary, protein content of the samples increased significantly with the increase in both irrigation and nitrogen doses (Fig 3). As is seen from the table, the highest protein level was recorded for the sample treated with 270 kg ha^-1^ nitrogen (11.15%).

**Fig 4:**
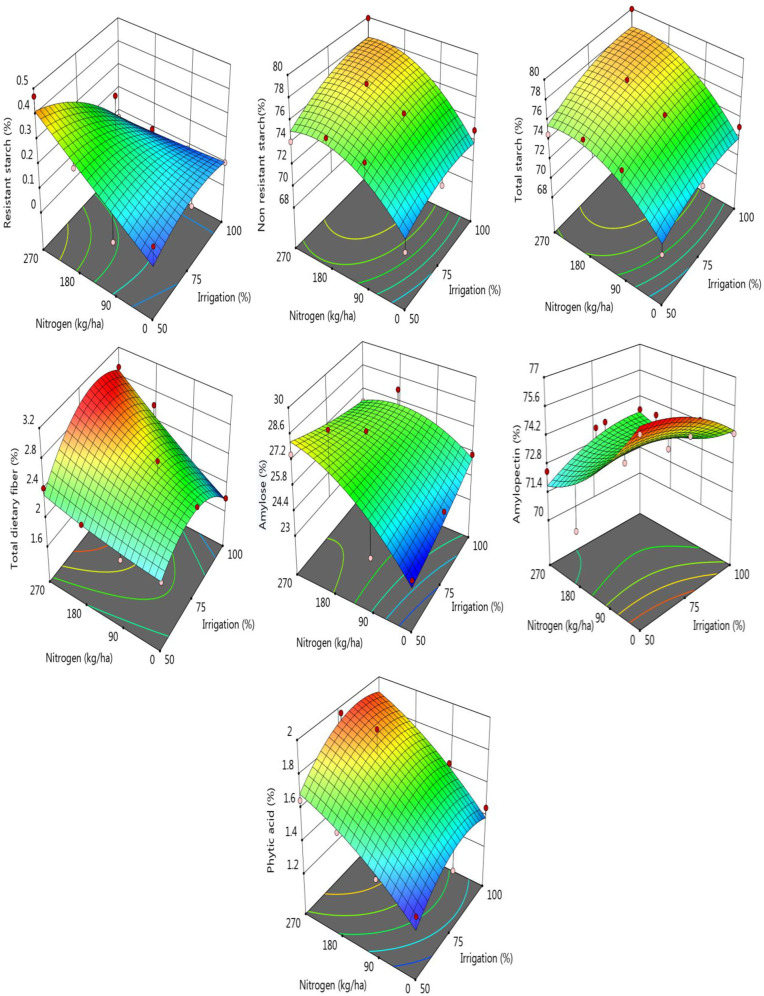
Change in some nutritional parameters of the samples according to the nitrogen-irrigation treatments.

Resistant starch content of the samples was affected by the irrigation significantly and it was monitored that the lowest RS content (0.08%) was determined for the sample irrigated as 100% and the highest RS was for the sorghum samples treated with 180 or 270 kg ha^-1^ nitrogen doses. Decrement in the RS caused an increase in the non-resistant starch. It was determined to be 73.7% and 75.84 for the irrigations of 50 and 100%, respectively. There was a negative correlation between RS and NRS contents of the sorghum samples treated with different irrigations. For the increased nitrogen doses, NRS also increased significantly (S2 File, Fig 4, p < 0.05), and it was recorded as 71.22% and 77.05 for the 180 and 270 kg ha^-1^ nitrogen doses, respectively. Total starch content of the samples ranged between 73.93–75.92% for the samples exposed to different irrigation levels and it was recorded that the increase in the irrigation level resulted in a significant increase for the total starch content (p < 0.05). For the samples exposed to different nitrogen doses, a similar and significant increment in the total starch content of the samples was also observed. Total starch level was determined as 71.33% for the samples treated with no nitrogen while the total starch content of the samples treated with 270 kg ha^-1^ nitrogen was found to be 77.29%. Irrigation treatment as 75% caused a significant increase in the phytic acid content of the samples from 1.49 to 1.70% and also an increase in the nitrogen doses caused a significant increase in the phytic acid content of the samples. For the total dietary fiber (TDF) content of the sorghum samples, the increase in the nitrogen doses from 0 to 270 kg ha^-1^ showed a significant increase compared to irrigation effects on the fiber level (Fig 4). Amylose and amylopectin fractions of the starch were also affected by the nitrogen treatment, and irrigation showed a little and insignificant effect on it. As is seen from Fig 4 and S2 File, increase in nitrogen doses caused a little decrement in the amylopectin and a significant increment in the amylose fractions of the sorghum starch components.

Ash content was influenced by irrigation, nitrogen dose, and their interaction. It decreased as irrigation and nitrogen doses increased. Based on irrigation levels, it ranged from 1.58% (I100) to 1.78% (I50), while based on nitrogen doses, it varied between 1.36% (N270) and 1.85% (N0). Regarding the interaction between irrigation and nitrogen dose, the highest ash content (2.14%) was observed at I50 and N0, whereas the lowest (1.08%) was recorded at I100 and N270.

**Fig 5:**
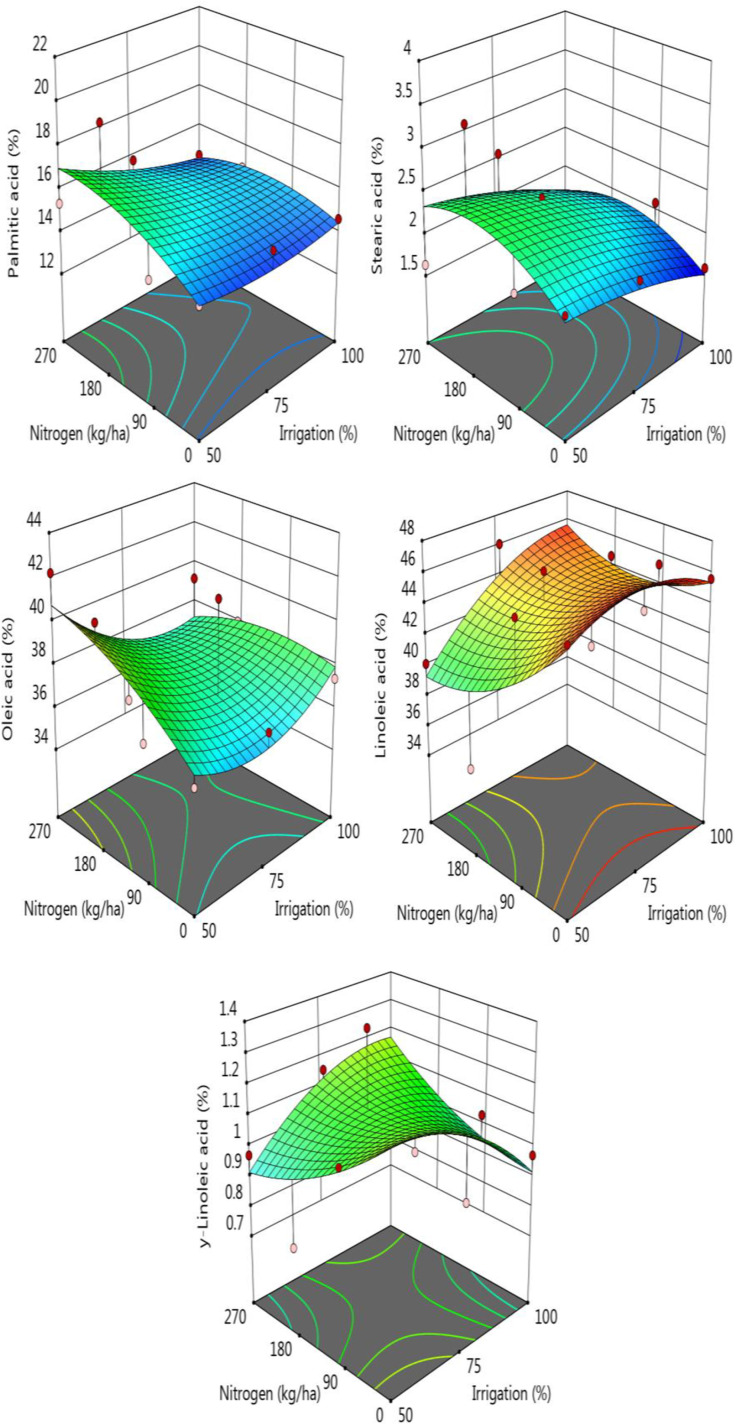
Change in major fatty acids of the samples according to the nitrogen-irrigation treatments.

Change in major fatty acid profile of the sorghum grains exposed to different irrigation levels and nitrogen doses were given in S3 File and Fig 5. For the sorghum samples, palmitic, stearic, oleic, linoleic and γ-linoleic acids were the major fatty acids detected in the samples. As is seen from Fig 5, palmitic acid was not affected by the irrigation and nitrogen, but a significant decrease and increase with the increase in irrigation and nitrogen levels was observed for the stearic acid levels, respectively. It was detected as 2.29% and 1.73% for the irrigation level of 50 and 100%, respectively while it was recorded as 1.86, 2.47 and 1.94% for 0, 18 and 27 kg nitrogen treatments, respectively. Oleic acid which is the second major fatty acids of the samples was significantly affected the processing variables, and it was in the range of 37.86–39.08% for the irrigation levels and 36.78–38.66 for the nitrogen doses. Increase in the nitrogen doses caused an increase in the oleic acid content of the samples (Fig 5). The level of linoleic acid which is the most abundant fatty acids increased significantly with the increase in irrigation and decreased with the increase of nitrogen level (Fig 5). It was also determined that both irrigation and nitrogen treatment showed no huge effect on the γ-linolenic acid levels of the sorghum samples (S3 File, Fig 5).

**Fig 6:**
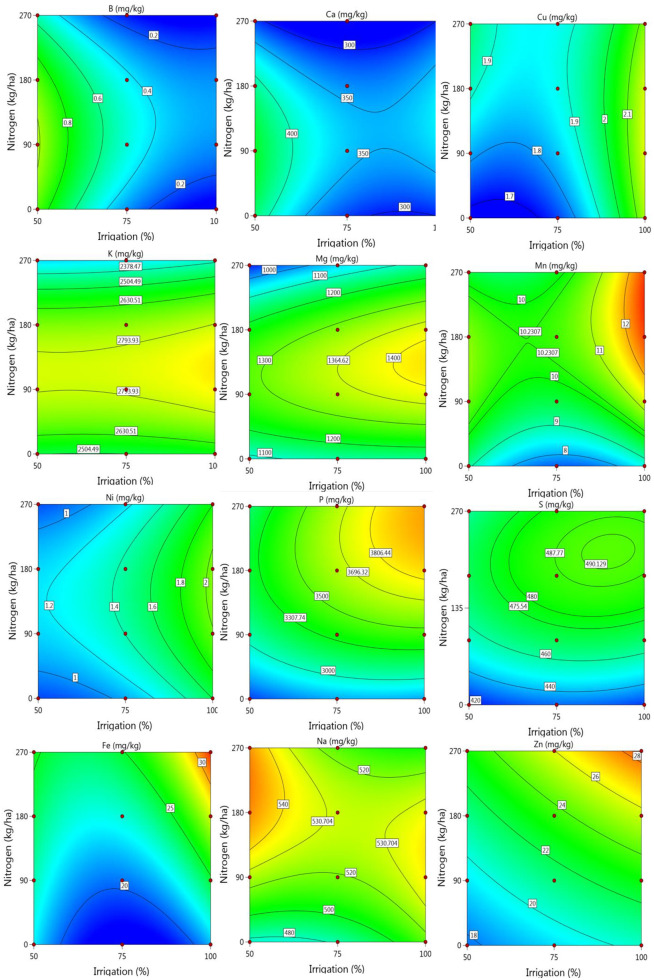
Change in some minerals of the samples according to the nitrogen-irrigation treatments.

Major and trace mineral of the sorghum samples exposed to different irrigation levels and nitrogen doses are tabulated in S4 File and Fig 6. As is seen from the table, Ca, K, Mg, N, P and S were the major elements detected in the samples and the others were the trace levels.

Different irrigation levels and nitrogen doses had significant effects on the mineral content of sorghum samples (S4 File, Fig 6). Remarkable changes were observed in the contents of major elements such as calcium (Ca), potassium (K), magnesium (Mg), phosphorus (P), nitrogen (N), and sulfur (S). With an increase in irrigation levels, calcium content showed a decreasing trend, dropping from 398.70 mg kg^-1^ at the 50% irrigation level to 334.68 at the 100% irrigation level. In contrast, potassium content increased from 2568.23 to 2642.54 mg kg^-1^, and magnesium content rose from 1124.41 to 1258.17 mg kg^-1^. The increase in nitrogen doses also had significant effects, with magnesium content reaching 1239.58 mg kg^-1^ at a nitrogen dose of 180 kg ha^-1^. At the microelement level, both irrigation and nitrogen applications had notable impacts on the iron (Fe) and zinc (Zn) contents. Specifically, Fe content was measured at 32.26 mg kg^-1^ and Zn content at 26.00 mg kg^-1^ under 100% irrigation and a nitrogen dose of 270 kg ha^-1^. While an increase in irrigation levels tended to reduce manganese (Mn) and boron (B) contents, higher nitrogen doses resulted in a significant increase in Mn levels. For example, Mn content was measured at 8.25 mg kg^-1^with 0 kg ha^-1^ nitrogen but increased to 10.90 mg kg^-1^ with 270 kg ha^-1^ nitrogen.

## Discussion

This study examined the effects of irrigation levels and nitrogen doses on the morphological characteristics, grain yield, and nutritional properties of sorghum. The interactions between irrigation and nitrogen applications were analyzed and compared with the responses of other crops.

The annual evapotranspiration (ET) rate of crop plants is typically substantially greater than the total precipitation received during their growing season [31]. Furthermore, previous studies have demonstrated a positive correlation between crop productivity and the ET rate [32]. Consistent with previous studies, while increased ET was found to enhance yield, the impact of nitrogen variation was also determined to be significant. Additionally, the CWP values observed were found to be similar to those reported by Jiang et al. [31] and Fang et al. [33].

The yield response factor (ky) is a crop-specific parameter that quantifies the reduction in relative yield resulting from decreased water consumption under abiotic stress conditions [34]. This parameter is also influenced by fertilization practices [35]. In this study, ky was affected by different nitrogen fertilization levels. The interaction between water availability and nitrogen nutrition was observed to have a significant impact on various traits assessed in sorghum.

Increases in irrigation levels and nitrogen doses were observed to enhance plant height, thousand-grain weight, and grain yield. Nitrogen fertilization accelerates growth and development in the meristem cells of internodes, resulting in elongated internodes, increased plant height, and improved biological yield. However, this increase is not constant; in cases of nitrogen deficiency, the growth period shortens, plant height decreases, and reductions in biomass and grain yield are observed [20]. Similarly, water deficiency leads to reduced photosynthesis and stomatal closure, resulting in shorter plant height, restricted root development, and decreases in biological and grain yields [36]. The loss in morphological characteristics due to reduced irrigation levels has been reported by Kale et al. [37]in maize and by Varol et al. (2020)[36] in chickpeas. Similarly, reductions due to nitrogen deficiency have been documented in wheat [38].

The study found that increasing irrigation levels resulted in higher amounts of protein, oil, resistant starch (RS), non-resistant starch (NRS), total starch, and phytic acid. While higher doses of nitrogen fertilization decreased oil content, a nitrogen dose of 180 kg per hectare produced the highest values for several characteristics. Both irrigation and nitrogen fertilization extended the grain filling period, leading to healthier grains with increased levels of oil, protein, starch, and minerals. Nitrogen fertilization increased grain photosynthesis and promoted starch accumulation during the grain-filling period, thereby enhancing grain growth and starch content [39]. However, the inability of nutrients accumulated in leaves to be transported to grains due to water stress resulted in decreased protein and starch content in grains [36]. Drought stress negatively affected total starch, amylose, and amylopectin content by impairing the activation of enzymes involved in starch synthesis [40].

Nitrogen is utilized in plants for chlorophyll and protein synthesis, and nitrogen fertilization increases protein and starch ratios [41]. Increased irrigation has been reported to enhance the resistant starch (RS) ratio in grains [1]. Yu et al. [40] observed that irrigation increased total starch and amylose content, as well as amylose/amylopectin ratios in barley. However, [42] reported that while irrigation could increase amylopectin and starch content, it decreased amylose/amylopectin ratios in wheat grains. Increased nitrogen fertilization levels have been found to raise amylose content and reduce amylopectin branching ratios in rice [16], wheat [38], and maize [13]. In contrast to other studies, Ovando-Martínez et al. -[43] reported that nitrogen fertilization increased amylose content under water stress in beans. Xia et al. [44]noted that water stress decreased RS content in wheat, while high nitrogen fertilizer levels increased it. Kaplan et al. [13] also reported an increase in resistant starch with nitrogen fertilization. Su et al. [45] stated that increasing nitrogen doses reduced the total phytic acid concentration in cereal grains. Higher nitrogen application has been shown to enhance ash content as a result of increased carbon assimilation [46]. Water stress limits root development, reducing ash content, and increasing fiber content, but not affecting lipids [47]. Water stress also increases fiber content in cereal grains by enhancing cell wall components, while reducing protein and oil content [36].

Kaplan et al. [48] reported that irrigation did not affect oil content in popcorn, while Pavlista et al. [49] found similar results in canola. However, Keshavarz Afshar et al. [50] in milk thistle, Pavlista et al. [51] in camelina, and Kaplan et al. [52] in maize reported an increase in oil content, consistent with our study. Akcura et al. [53] observed the highest oil content at a 75% irrigation level in different peanut varieties. Sebei et al. [54] examined four different levels of nitrogen application (none, low, medium, and high nitrogen fertilization) in canola and found that high nitrogen doses reduced the oil content of canola seeds. In our study, an increase in nitrogen dose resulted in a decrease in oil content and an increase in dietary fiber content. Irrigation water levels are a significant factor affecting oleic acid content [53]. Variations in linoleic acid content, influenced by genetics and agricultural practices such as irrigation and fertilization, directly impact oleic acid content. Research indicates that an increase in linoleic acid content results in a decrease in oleic acid content [55].

In this study, the highest linoleic acid content was found under 100% irrigation combined with 0 and 9 kg/ha nitrogen applications. The interaction of these factors revealed that linoleic acid levels were also high at a nitrogen application of 50 kg/ha under both 75% and 100% irrigation. These findings suggest that irrigation has a more significant impact on linoleic acid content than nitrogen application.

Pavlista et al. [49] investigated the fatty acid composition of camelina grown under four different irrigation levels and reported that linolenic acid, the primary component, increased with the level of irrigation. However, in our study, increasing the nitrogen fertilizer dose resulted in a reduction of oil content. In contrast, Lai et al. [56] found that optimal nitrogen application significantly increased the overall fatty acid content, including total unsaturated fatty acids, linoleic acid, and oleic acid. Additionally, Ashraf et al. [50] reported that nitrogen fertilization did not affect the linoleic acid content in black cumin oil. These studies indicate that plants respond differently to variations in oil and fatty acid content.

In the study, increased irrigation levels had positive effects on Cu, Fe, Mg, Mn, Ni, P, and Zn elements, while causing a decrease in other elements. Among nitrogen applications, 90 kg/ha N application represented the optimum level for most elements, while 180 and 270 kg/ha N applications resulted in the highest values for certain elements. Water stress can restrict plants’ uptake of mineral elements from the soil, leading to a decrease in mineral content in grains [57]. The uptake of many elements decreases with reduced irrigation levels; however, water stress affects the uptake rates of soil elements at different levels [58]. The Fe and Zn content in grains has been reported to be more influenced by environmental factors such as irrigation and fertilization rather than genetic traits. Drought stress reduces the total P content in plants and negatively affects plant growth and development [59]. Nitrogen fertilization increases the S, N, P, and K content in cereal grains [60]. Plant S requirements and metabolism are closely related to N levels, with a positive and significant relationship between them [61].

Nitrogen fertilization increases the uptake of Fe and Zn by plants from the soil and enhances their content in cereal grains [62]. Calcium plays a protective role in plants under stress conditions. It influences various structural and physiological processes such as membrane structure, cell wall formation, cell division, and photomorphogenesis in plants [63]. Soil moisture deficiency has been reported to significantly reduce Mg content in grains [64]. A decrease in Mg levels negatively affects photosynthetic activity, grain formation, and grain characteristics [65]. The increases in Zn, Fe, K, Mg, and P content with higher irrigation and nitrogen fertilization levels are consistent with studies conducted on maize [48], wheat [66], and teff [67].

## Conclusion

This study examines how different levels of irrigation and nitrogen application affect the yield, yield components, and nutritional properties of sorghum grain. The results indicated that applying 100% of the irrigation water in combination with nitrogen doses ranging from 180 to 270 kg per hectare is effective in balancing both yield and quality traits, including protein, oil, starch, and mineral content. Future research involving various sorghum varieties across different climatic and soil conditions will further validate these findings and help develop more comprehensive recommendations.

## Supporting information

S1 File Change in seed yield and some morphological parameters of the samples according to the nitrogen-irrigation treatments. (DOCX)

S2 File Change in some biochemical characteristics of the samples according to the nitrogen-irrigation treatments. (DOCX)

S3 File Change in major fatty acids of the samples according to the nitrogen-irrigation treatments. (DOCX)

S4 File Change in some minerals of the samples according to the nitrogen-irrigation treatments. (DOCX)
