# Supplementary material for: Morphological and nutritional responses of sorghum to variable irrigation levels and nitrogen doses
Source: PLoS One. 2025 Jun 2;20(5):e0323901. doi: 10.1371/journal.pone.0323901 (PMC12129201; doi:10.1371/journal.pone.0323901)
Supplement: S1 File — (DOCX) [file pone.0323901.s001.docx]

**Supplementary Information 1.** Change in seed yield and some morphological parameters of the samples according to the nitrogen-irrigation treatments

| Application | Irrigation (%) | PH | TSW | NGPP | WGPP | SY |
| --- | --- | --- | --- | --- | --- | --- |
| Irrigation Level | 50 | 157.46^b^ | 31.41 | 3221.85^c^ | 109.45^b^ | 4011^c^ |
|  | 75 | 155.04^b^ | 31.73 | 3675.57^b^ | 127.06^a^ | 4964^b^ |
|  | 100 | 180.50^a^ | 31.02 | 4197.97^a^ | 136.57^a^ | 5906^a^ |
|  | N (kg ha^-1^) | PH | TSW | NGPP | WGPP | S Y |
| Nitrogen Doses | 0 | 162.22 | 30.50^b^ | 2962.81^d^ | 96.52^d^ | 3573^d^ |
|  | 90 | 161.83 | 31.51^b^ | 3854.91^b^ | 129.92^b^ | 4931^c^ |
|  | 180 | 168.00 | 32.91^a^ | 4552.42^a^ | 157.60^a^ | 6071^a^ |
|  | 270 | 165.27 | 30.64^b^ | 3423.72^c^ | 113.42^c^ | 5265^b^ |
|  |  |  |  |  |  |  |
| Irrigation (%) | N (kg ha^-1^) | PH | TSW | NGPP | WGPP | SY |
| 50 | 0 | 164.83^ab^ | 31.94^abcd^ | 2162.18^e^ | 73.37^f^ | 2496^f^ |
| 50 | 90 | 161.83^b^ | 31.16^abcde^ | 3417.29^bcd^ | 118.06^cde^ | 4172^de^ |
| 50 | 180 | 154.83^b^ | 31.56^abcd^ | 4160.32^b^ | 144.10^abc^ | 4842^c^ |
| 50 | 270 | 148.33^b^ | 30.96^bcde^ | 3147.62^cd^ | 102.27^ef^ | 4534^cd^ |
| 75 | 0 | 159.17^b^ | 29.92^cde^ | 3518.26^bcd^ | 110.31^cde^ | 3718^e^ |
| 75 | 90 | 149.00^b^ | 30.87^bcde^ | 4020.03^bc^ | 134.48^bcde^ | 4748^cd^ |
| 75 | 180 | 167.33^b^ | 33.80^a^ | 4170.37^b^ | 155.25^ab^ | 6253^b^ |
| 75 | 270 | 154.67^b^ | 32.32^abcd^ | 2993.62^de^ | 108.24^def^ | 5137^c^ |
| 100 | 0 | 162.67^b^ | 29.63^de^ | 3208.00^cd^ | 105.87^def^ | 4504^cd^ |
| 100 | 90 | 174.67^ab^ | 32.49^abc^ | 4127.41^b^ | 137.23^bcd^ | 5875^b^ |
| 100 | 180 | 191.83^a^ | 33.35^ab^ | 5326.55^a^ | 173.44^a^ | 7120^a^ |
| 100 | 270 | 192.83^a^ | 28.63^e^ | 4129.92^b^ | 129.74^bcde^ | 6125^b^ |

**PH:** plant height (cm); **TSW:** thousand seed weight (g); **NGPP:** number of grains per panicle; **WGPP:** weight of grains per panicle (g); **SY:** seed yield (kg ha^-1^)
