# Supplementary material for: Morphological and nutritional responses of sorghum to variable irrigation levels and nitrogen doses
Source: PLoS One. 2025 Jun 2;20(5):e0323901. doi: 10.1371/journal.pone.0323901 (PMC12129201; doi:10.1371/journal.pone.0323901)
Supplement: S2 File — (DOCX) [file pone.0323901.s002.docx]

**Supplementary Information 2.** Change in some biochemical characteristics of the samples according to the nitrogen-irrigation treatments

| Application | Irrigation (%) | Oil | Protein | Ash | Resistant Starch | Non- Resistant Starch | Total Starch | Phytic acid | Total Dietary Fiber | Amylose | Amylopectin |
| --- | --- | --- | --- | --- | --- | --- | --- | --- | --- | --- | --- |
| Irrigation | 50 | 5.01^b^ | 9.71^b^ | 1.78^a^ | 0.24^a^ | 73.70 | 73.93 | 1.49^b^ | 2.18^b^ | 26.13 | 73.87 |
|  | 75 | 5.87^a^ | 10.75^a^ | 1.68^ab^ | 0.21^a^ | 75.60 | 75.81 | 1.70^a^ | 2.64^a^ | 26.13 | 73.96 |
|  | 100 | 5.43^ab^ | 10.84^a^ | 1.58^b^ | 0.08^b^ | 75.84 | 75.92 | 1.67^a^ | 2.31^b^ | 26.44 | 73.55 |
|  | N  (kg ha^-1^) | Oil | Protein | Ash | Resistant Starch | Non- Resistant Starch | Total Starch | Phytic acid | Total Dietary Fiber | Amylose | Amylopectin |
| Nitrogen | 0 | 6.60^a^ | 9.47^c^ | 1.85^a^ | 0.11^bc^ | 71.22^b^ | 71.33^b^ | 1.36^c^ | 2.06^b^ | 24.67^c^ | 75.33^a^ |
|  | 90 | 6.06^a^ | 10.33^b^ | 1.79^a^ | 0.10^c^ | 75.63^a^ | 75.73^a^ | 1.54^bc^ | 2.21^b^ | 24.97^bc^ | 75.03^ab^ |
|  | 180 | 4.92^b^ | 10.81^ab^ | 1.71^a^ | 0.25^a^ | 76.28^a^ | 76.53^a^ | 1.75^ab^ | 2.52^a^ | 28.35^a^ | 71.65^c^ |
|  | 270 | 4.18^b^ | 11.15^a^ | 1.36^b^ | 0.24^ab^ | 77.05^a^ | 77.29^a^ | 1.83^a^ | 2.72^a^ | 26.95^ab^ | 73.18^bc^ |
|  |  |  |  |  |  |  |  |  |  |  |  |
| Irrigation (%) | N  (kg ha^-1^) | Oil | Protein | Ash | Resistant Starch | Non- Resistant Starch | Total Starch | Phytic acid | Total Dietary Fiber | Amylose | Amylopectin |
| 50 | 0 | 6.38^ab^ | 9.40^e^ | 2.14^a^ | 0.14^bc^ | 69.29^c^ | 69.43^c^ | 1.32^c^ | 2.05^cde^ | 23.59^b^ | 76.41^a^ |
| 50 | 90 | 5.37^abcd^ | 9.78^cde^ | 1.73^ab^ | 0.06^c^ | 75.38^abc^ | 75.44^abc^ | 1.41^bc^ | 2.03^de^ | 23.76^b^ | 76.24^a^ |
| 50 | 180 | 4.27^cd^ | 9.82^cde^ | 1.77^ab^ | 0.27^abc^ | 75.96^abc^ | 76.23^abc^ | 1.57^abc^ | 2.21^bcde^ | 29.64^a^ | 70.36^b^ |
| 50 | 270 | 4.03^d^ | 9.88^cde^ | 1.48^bc^ | 0.47^a^ | 74.15^abc^ | 74.63^abc^ | 1.65^abc^ | 2.42^abcde^ | 27.54^ab^ | 72.46^ab^ |
| 75 | 0 | 6.78^a^ | 9.56^cde^ | 1.68^ab^ | 0.10^bc^ | 71.34^bc^ | 71.44^bc^ | 1.31^c^ | 2.34^abcde^ | 24.75^b^ | 75.25^a^ |
| 75 | 90 | 6.46^a^ | 10.23^bcde^ | 1.76^ab^ | 0.16^bc^ | 76.34^ab^ | 76.49^ab^ | 1.58^abc^ | 2.68^abcd^ | 25.45^ab^ | 75.55^ab^ |
| 75 | 180 | 5.96^abc^ | 11.52^ab^ | 1.76^ab^ | 0.39^ab^ | 77.64^ab^ | 78.03^ab^ | 1.95^a^ | 2.71^abc^ | 27.51^ab^ | 72.49^ab^ |
| 75 | 270 | 4.27^cd^ | 11.72^ab^ | 1.53^bc^ | 0.19^abc^ | 77.09^ab^ | 77.28^ab^ | 1.96^a^ | 2.83^ab^ | 26.82^ab^ | 73.56^ab^ |
| 100 | 0 | 6.64^a^ | 9.45^de^ | 1.74^ab^ | 0.10^bc^ | 73.02^abc^ | 73.12^abc^ | 1.45^bc^ | 1.79^e^ | 25.68^ab^ | 74.32^ab^ |
| 100 | 90 | 6.33^ab^ | 10.97^abcd^ | 1.88^ab^ | 0.08^c^ | 75.18^abc^ | 75.26^abc^ | 1.63^abc^ | 1.91^e^ | 25.71^ab^ | 74.29^ab^ |
| 100 | 180 | 4.52^bcd^ | 11.09^abc^ | 1.61^b^ | 0.09^c^ | 75.24^abc^ | 75.32^abc^ | 1.72^abc^ | 2.62^abcd^ | 27.91^ab^ | 72.08^ab^ |
| 100 | 270 | 4.24^cd^ | 11.85^a^ | 1.08^c^ | 0.07^c^ | 79.91^a^ | 79.97^a^ | 1.88^ab^ | 2.93^a^ | 26.48^ab^ | 73.52^ab^ |
