# Supplementary material for: Morphological and nutritional responses of sorghum to variable irrigation levels and nitrogen doses
Source: PLoS One. 2025 Jun 2;20(5):e0323901. doi: 10.1371/journal.pone.0323901 (PMC12129201; doi:10.1371/journal.pone.0323901)
Supplement: S3 File — (DOCX) [file pone.0323901.s003.docx]

**Supplementary Information 3.** Change in major fatty acids of the samples according to the nitrogen-irrigation treatments

| Application | Irrigation (%) | Palmitic Acid  (C16:0) | Stearic Acid  (C18:0) | Oleic Acid  (C18:1n9c) | Linoleic Acid  (C18:2n6c) | y-Linolenic Acid  (C18:3n6) |
| --- | --- | --- | --- | --- | --- | --- |
| Irrigation | 50 | 16.06 | 2.29^a^ | 39.08^a^ | 41.69^b^ | 1.05 |
|  | 75 | 15.02 | 2.10^ab^ | 37.10^b^ | 44.78^a^ | 1.11 |
|  | 100 | 14.69 | 1.73^b^ | 37.86^ab^ | 44.79^a^ | 1.02 |
|  | N  (kg ha^-1^) | Palmitic Acid  (C16:0) | Stearic Acid  (C18:0) | Oleic Acid  (C18:1n9c) | Linoleic Acid  (C18:2n6c) | y-Linolenic Acid  (C18:3n6) |
| Nitrogen | 0 | 14.71^b^ | 1.86^b^ | 36.78 | 45.66^a^ | 1.10 |
|  | 90 | 14.33^b^ | 1.89^b^ | 38.43 | 44.41^ab^ | 1.04 |
|  | 180 | 16.79^a^ | 2.47^a^ | 38.19 | 41.67^c^ | 1.03 |
|  | 270 | 15.19^ab^ | 1.94^ab^ | 38.66 | 43.27^bc^ | 1.07 |
|  |  |  |  |  |  |  |
| Irrigation (%) | N  (kg ha^-1^) | Palmitic Acid  (C16:0) | Stearic Acid  (C18:0) | Oleic Acid  (C18:1n9c) | Linoleic Acid  (C18:2n6c) | y-Linolenic Acid  (C18:3n6) |
| 50 | 0 | 14.54^b^ | 2.05^b^ | 36.27^de^ | 45.79^a^ | 1.38^a^ |
| 50 | 90 | 14.32^b^ | 1.95^b^ | 36.84^cde^ | 45.93^a^ | 1.09^ab^ |
| 50 | 180 | 20.04^a^ | 3.53^a^ | 40.99^abc^ | 34.90^c^ | 0.75^c^ |
| 50 | 270 | 15.33^b^ | 1.63^b^ | 42.21^a^ | 40.12^b^ | 0.97^bc^ |
| 75 | 0 | 14.99^b^ | 1.93^b^ | 36.70^cde^ | 45.62^a^ | 0.94^bc^ |
| 75 | 90 | 13.89^b^ | 1.61^b^ | 41.54^ab^ | 42.01^ab^ | 1.02^bc^ |
| 75 | 180 | 15.43^b^ | 2.29^b^ | 35.59^de^ | 45.47^a^ | 1.35^a^ |
| 75 | 270 | 15.77^ab^ | 2.56^ab^ | 34.57^e^ | 46.03^a^ | 1.15^ab^ |
| 100 | 0 | 14.61^b^ | 1.59^b^ | 37.36^bcde^ | 45.59^a^ | 0.97^bc^ |
| 100 | 90 | 1478^b^ | 2.09^b^ | 36.90^cde^ | 45.28^a^ | 1.03^bc^ |
| 100 | 180 | 14.89^b^ | 1.59^b^ | 37.98^abcde^ | 44.63^a^ | 0.99^bc^ |
| 100 | 270 | 14.48^b^ | 1.64^b^ | 39.19^abcd^ | 43.66^ab^ | 1.09^ab^ |
