# Supplementary material for: Morphological and nutritional responses of sorghum to variable irrigation levels and nitrogen doses
Source: PLoS One. 2025 Jun 2;20(5):e0323901. doi: 10.1371/journal.pone.0323901 (PMC12129201; doi:10.1371/journal.pone.0323901)
Supplement: S4 File — (DOCX) [file pone.0323901.s004.docx]

**Supplementary Information 4.** Change in some minerals of the samples according to the nitrogen-irrigation treatments

| Application | Irrigation (%) | B | Ca | Cu | Fe | K | Mg | Mn | Na | Ni | P | S | Zn |
| --- | --- | --- | --- | --- | --- | --- | --- | --- | --- | --- | --- | --- | --- |
| Irrigation Level | 50 | 0.84^a^ | 398.70^a^ | 1.84^b^ | 23.67^b^ | 2568.23 | 1124.41 | 10.04^b^ | 525.86 | 0.99^b^ | 3006.79^b^ | 450.92 | 20.32^b^ |
|  | 75 | 0.33^b^ | 316.47^b^ | 1.89^b^ | 21.46^c^ | 2580.36 | 1205.22 | 9.35^b^ | 511.26 | 1.26^b^ | 3350.59^ab^ | 467.18 | 22.51^ab^ |
|  | 100 | 0.18^b^ | 334.68^b^ | 2.16^a^ | 26.46^a^ | 2642.54 | 1258.17 | 11.30^a^ | 523.29 | 1.85^a^ | 3495.49^a^ | 465.98 | 24.35^a^ |
|  | N (kg ha^-1^) | B | Ca | Cu | Fe | K | Mg | Mn | Na | Ni | P | S | Zn |
| Nitrogen Doses | 0 | 0.38 | 332.71^b^ | 1.86^b^ | 20.73^b^ | 2471.29^b^ | 1090.48^bc^ | 8.25^b^ | 496.01^b^ | 1.12 | 2699.53^b^ | 427.27^c^ | 18.94^c^ |
|  | 90 | 0.69 | 430.15^a^ | 1.93^ab^ | 21.59^b^ | 2972.66^a^ | 1404.84^a^ | 10.90^a^ | 513.03^ab^ | 1.63 | 3250.16^a^ | 453.79^bc^ | 21.61^bc^ |
|  | 180 | 0.45 | 333.99^b^ | 2.08^a^ | 26.20^a^ | 2596.80^b^ | 1239.58^ab^ | 10.78^a^ | 552.94^a^ | 1.42 | 3614.48^a^ | 491.12^a^ | 23.02^ab^ |
|  | 270 | 0.29 | 302.94^b^ | 1.98^ab^ | 26.92^a^ | 2347.43^b^ | 1048.85^c^ | 10.90^a^ | 518.57^ab^ | 1.31 | 3572.99^a^ | 473.27^b^ | 26.00^a^ |
|  |  |  |  |  |  |  |  |  |  |  |  |  |  |
| Irrigation (%) | N (kg ha^-1^) | B | Ca | Cu | Fe | K | Mg | Mn | Na | Ni | P | S | Zn |
| 50 | 0 | 0.55^ab^ | 331.08^b^ | 1.70^b^ | 21.42^cde^ | 2493.20^abc^ | 1063.04^bcd^ | 8.76^bc^ | 438.04^b^ | 1.18^b^ | 2457.17^d^ | 416.74^c^ | 16.23^c^ |
| 50 | 90 | 1.48^a^ | 634.89^a^ | 1.85^b^ | 21.98^cde^ | 2713.18^ab^ | 1228.77^abcd^ | 11.77^ab^ | 569.07^a^ | 1.24^b^ | 3124.78^abcd^ | 458.92^bc^ | 20.60^bc^ |
| 50 | 180 | 0.82^ab^ | 338.41^b^ | 1.90^b^ | 27.77^b^ | 2553.41^abc^ | 1131.04^bcd^ | 10.07^abc^ | 566.65^a^ | 0.87^b^ | 3271.37^abcd^ | 472.79^abc^ | 22.76^abc^ |
| 50 | 270 | 0.49^b^ | 290.44^b^ | 1.91^ab^ | 23.49^cd^ | 2513.13^abc^ | 1074.79^bcd^ | 9.56^bc^ | 529.69^ab^ | 0.68^b^ | 3173.84^abcd^ | 455.24^bc^ | 21.70^abc^ |
| 75 | 0 | 0.46^b^ | 336.58^b^ | 1.74^b^ | 18.73^e^ | 2581.82^abc^ | 1260.78^abcd^ | 6.95^c^ | 508.70^ab^ | 0.74^b^ | 2723.87^cd^ | 421.43^c^ | 19.51^bc^ |
| 75 | 90 | 0.47^b^ | 316.70^b^ | 1.77^b^ | 19.82^de^ | 3137.24^a^ | 1420.12^ab^ | 9.87^abc^ | 484.67^ab^ | 0.78^b^ | 3411.62^abcd^ | 446.53^bc^ | 22.10^abc^ |
| 75 | 180 | 0.31^b^ | 306.63^b^ | 1.74^b^ | 22.26^cde^ | 2589.93^abc^ | 1244.57^abcd^ | 10.50^ab^ | 546.21^a^ | 1.70^ab^ | 3953.51^ab^ | 543.75^a^ | 21.81^abc^ |
| 75 | 270 | 0.09^b^ | 305.96^b^ | 2.09^ab^ | 25.01^bc^ | 2012.46^c^ | 895.41^d^ | 10.08^abc^ | 505.47^ab^ | 1.84^ab^ | 3313.35^abcd^ | 457.00^bc^ | 26.62^ab^ |
| 100 | 0 | 0.12^b^ | 330.48^b^ | 2.14^ab^ | 22.05^cde^ | 2338.83^bc^ | 947.61^cd^ | 9.06^bc^ | 541.28^a^ | 1.44^b^ | 2917.54^bcd^ | 443.64^bc^ | 21.08^bc^ |
| 100 | 90 | 0.11^b^ | 338.86^b^ | 2.18^ab^ | 22.97^cde^ | 3067.55^a^ | 1565.63^a^ | 11.33^ab^ | 485.34^ab^ | 2.88^a^ | 3214.06^abcd^ | 455.93^bc^ | 22.14^abc^ |
| 100 | 180 | 0.22^b^ | 356.94^b^ | 2.38^a^ | 28.57^ab^ | 2647.05^abc^ | 1343.11^abc^ | 11.78^ab^ | 545.96^a^ | 1.69^ab^ | 3618.56^abc^ | 456.85^bc^ | 24.50^abc^ |
| 100 | 270 | 0.21^b^ | 312.43^b^ | 1.95^ab^ | 32.26^a^ | 2516.71^abc^ | 1176.34^abcd^ | 13.05^a^ | 520.55^ab^ | 1.41^b^ | 4231.77^a^ | 507.49^ab^ | 29.68^a^ |
